# Positive Impact of Vaccinal Status Among Notified Measles Cases in Romania in 2020–2024

**DOI:** 10.3390/epidemiologia6040063

**Published:** 2025-10-11

**Authors:** Valerian-Ionuț Stoian, Iulia Chiscop, Aurora Stănescu, Mariana Daniela Ignat, Raisa Eloise Barbu, Mădălina Nicoleta Matei, Alexia Anastasia Ștefania Baltă, Liliana Baroiu, Iulia Draghiev, Mihaela Debita

**Affiliations:** 1 Medical Department, Faculty of Medicine & Pharmacy, ‘Dunărea de Jos’ University of Galați, 800008 Galați, Romania; valerian.stoian@ugal.ro (V.-I.S.); iuliadraghiev@yahoo.com (I.D.); mdebita@ugal.ro (M.D.); 2 National Institute for Public Health, 050463 Bucharest, Romania; 3 Department of Clinical Surgery, Faculty of Medicine and Pharmacy, “Dunarea de Jos” University, 800008 Galati, Romania; 4 Medicine Faculty, ‘Carol Davila’ University of Medicine & Pharmacy, 050474 Bucharest, Romania; 5 Department of Clinical Medicine, Faculty of Medicine and Pharmacy, “Dunarea de Jos” University, 800008 Galati, Romania; mariana_daniela52@yahoo.com (M.D.I.); raisauibariu@gmail.com (R.E.B.); alexiaanastasia1998@yahoo.com (A.A.Ș.B.); lilibaroiu@yahoo.com (L.B.); 6 Department of Dental Medicine, Faculty of Medicine and Pharmacy, “Dunarea de Jos” University, 800008 Galati, Romania; madalina.matei@ugal.ro

**Keywords:** measles, vaccination, disease burden, Romania

## Abstract

Background and Objectives: Measles is a highly contagious but vaccine-preventable disease with significant morbidity in the European region, including Romania, especially in the post-COVID-19 era with low vaccination rates which no longer provide herd immunity. The current study aims to show how vaccination reduces the disease burden. Methods: A study using 29,148 cases with measles-compatible features in Romania from the 2020–2024 period was performed, analyzing symptoms, complications, and hospitalization rates comparatively between vaccinated and non-vaccinated groups. Results: Our findings show substantial hospitalization rates reduction among vaccinated cases with an over 12% decrease—depending on the number of MMR doses—as well as reduced severity of clinical features, but no significant effect on disease duration. Conclusions: MMR vaccination provides protection beyond primary disease prevention, as it reduces the disease burden among measles cases by reducing disease-related hospitalizations and improving clinical outcomes.

## 1. Introduction

Vaccines represent a significant breakthrough in public health through their ability to reduce disease burden, either by primary prevention, herd immunity, or improving outcomes in those contracting the disease [1]. Evaluating vaccine effectiveness and its impact on disease burden is an ongoing, orchestrated effort by all countries in the European Union under the coordination of the European Center for Disease Prevention and Control [2], and it is also a research target for numerous other studies. For example, pneumococcal vaccination in older people has been shown to protect against cardiovascular diseases [3].

In Europe, measles resurfaced in 2020 due to low MMR vaccine coverage caused by the population’s concerns toward the vaccine’s safety and effectiveness, as well as mistrust in medical information and experts, with a low perceived risk of measles [4]. COVID-19 vaccines contributed at least partly to the misplaced negative attitudes vaccines due to the population’s high expectations of preventing not only severe disease profiles and complications but also the disease itself. In reality, the protection was against specific SARS-CoV-2 strains with limited duration, allowing for breakthrough infections which, in turn, led those vaccinated who developed COVID-19 to question the validity of vaccination programs [5]. In Jordan, a surprising number of parents would rather let their children acquire measles immunity through natural infection rather than through the MMR vaccine, further showing the dwindling trust in vaccination campaigns worldwide [6].

Romania’s national vaccination schedule provides two MMR doses for measles: one at 12 months and another at age 5, in accordance with a Health Ministry Order [7]. The vaccination is mandatory through the general practitioner with the expenses fully covered by the Romanian government; however, there are no legal consequences for vaccination refusal. Moreover, starting with the 2016 outbreak, a third MMR dose was introduced for children aged 9–11 months. Although not part of the national vaccination schedule, it is also provided for free and regulated by the Health Ministry in the order declaring the outbreak, with the last one declared in December 2023 [8]. To promote vaccination, parents are also reminded to vaccinate their children once they reach an eligible age through automated text messages sent by the National Electronic Vaccination Registry. Measles vaccination was introduced in Romania in 1979 for the 9–12-month-old group, with age contingents tracing back to children born starting in 1974, while a second dose was recommended for children starting primary school since 1994. In 2004, the combined MMR vaccine started replacing the measles monovalent vaccine. Immediately after the vaccine introduction, the effect on disease burden was profound, even when using just one dose, with the incidence more than six times lower in 1980 compared to 1978 [9].

Although ideally, very high vaccine coverage is both desirable and required to obtain the herd immunity, the population’s reticence toward vaccination programs, especially in the context of how COVID-19 vaccination schedules were approached, has led to a steady declining vaccine coverage for other vaccine-preventable diseases, including measles. This phenomenon is severely apparent in Romania where in less than five years, MMR coverage decreased from almost 90% for the first dose in 2019 to less than 80% in 2023, with the second dose faring much worse, dropping from 80% in 2018 to almost 60% in 2023 [9].

The main reasons for non-vaccination in Romania have been previously identified and consist of failure to attend the vaccinator’s office, temporary medical contraindications, or plain MMR vaccination refusal, with the first mentioned reason being the most common and statistically significant [10].

In such circumstances, herd immunity is lost, potentially leading to a more severe disease burden, including higher hospitalization rates [11]. The most vulnerable population consists of children under the age of six who are not yet eligible for the full vaccination scheme and among whom measles incidence tends to be the highest [9].

## 2. Materials and Methods

A retrospective cohort study was conducted on 29,148 valid entries encompassing cases with measles-compatible clinical features from the national surveillance-based case series which were notified in Romania between 1 January 2020 and 31 December 2024, using data provided by the National Institute for Public Health of Romania, with the study being approved by the ethics committee of the University ‘Dunărea de Jos’ Galați.

The national measles database is maintained by the National Center for Communicable Diseases Surveillance and Control. Both this center and the regional centers are part of the Romanian National Institute for Public Health and play crucial roles in populating the database. The measles database contains field-acquired data by the Public Health Directorates through medical practitioners who suspect measles based on clinical features. The submitted form contains demographic- and disease-related information and strongly adheres to the General Data Protection Regulation.

Measles is one of the obligatory reportable communicable disease in Romania, and the information flux is regulated through disease-specific methodology, with the last version updated in 2024 [12]. The case definition adheres to the European Union decision 945/2018 [13]. For confirmation, either elevated measles-specific IgM in patients’ serum starting on day 5 after disease onset or PCR testing from nasopharyngeal swabs in the first few days is used. The dataset includes mostly confirmed cases (18,853) and probable cases (6650) based on clinical and epidemiological criteria. Both confirmed and probable cases amount to 87.6% of the cases analyzed. Of the confirmed cases, 14,645 (77.68%) tested positive for serum measles-specific IgM, while 4666 cases (24.75%) were confirmed using PCR from nasopharyngeal swabs. The overlapping 458 cases had both positive PCR and IgM results; the main reason for this phenomenon is that PCR specimens are sent to a regionally designated laboratory within the National Institute for Health network, which can take up to two weeks for a valid result, while IgM testing can also be performed locally by medical units with results usually being ready within 1–2 days. Possible cases based on clinical criteria alone are strongly discouraged, even more so during a national outbreak, but some parents, especially from minority communities, refuse to cooperate for further investigations or specimen retrieval. There are also cases where initial testing led to equivocal results and parents or patients refused a second blood test.

The dataset was explored using Python v3.13 (matplotlib v3.10.1 and Seaborn v0.13.2), which offers robust data scalability and adequate large-volume data management [14]. The data were split into vaccinated, non-vaccinated, and unknown MMR vaccine status groups. Reasons for unknown vaccination data vary but most commonly relate to the patient’s birth date predating the introduction of the National Electronic Vaccination Registry and the lack of relevant information from the patient’s general practitioner.

## 3. Results

**Figure 1:**
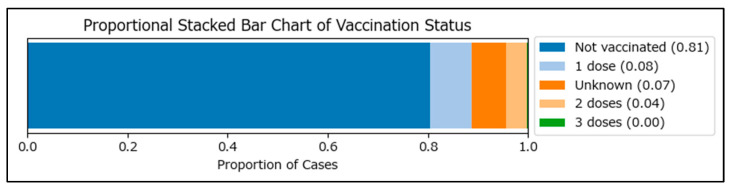
Stacked bar chart of the vaccination status of measles cases in Romania during the January 2020–December 2024 period.

Initial data segmentation identified 3674 vaccinated (2404 with one MMR dose, 1219 with two MMR doses, and 51 with three MMR doses), 23,468 non-vaccinated, and 2006 cases with an unknown vaccination status among measles cases in Romania during the January 2020–December 2024 period (Figure 1).

Consequently, cases were split based on living environment and vaccination status, as shown in Table 1. Urban areas were expected to show higher vaccination rates due to easier access to medical services. Instead, populations from rural areas showed higher vaccination coverage across all MMR doses, especially in the most important category of two doses.

The higher vaccination rates in rural areas are consistent with the lower access to health information in the rural areas, as previously described in other studies [15]. In this case, this works out in our favor, as the population is more likely to follow their doctor’s recommendations without further research. This is consistent with the previous findings provided by the Romanian National Institute for Public Health, showing that individuals from rural areas have a lower percentage of failure to show up for vaccination (35.8% for rural vs. 43.9% for urban) [16]. A chi-square test was performed for the living environment, showing a highly significant association between vaccination status and living environment (chi2 = 98.6, *p* < 0.001), suggesting that these findings are significant.

A one-way ANOVA was performed to assess differences in age among vaccinated, non-vaccinated, and unknown MMR vaccination status groups. Levene’s test for homogeneity of variances was highly significant (statistic 553.0905, *p*-value < 0.001), indicating that variances are not equal across groups. The ANOVA test also showed a highly significant result (statistic 1233.669, *p*-value ≈ 0), meaning that there are statistically significant differences in mean age between vaccination groups (non-vaccinated, vaccinated with at least one MMR dose, and unknown status). These findings suggest that age distributions differ substantially among vaccinated, non-vaccinated, and unknown status groups.

**Figure 2:**
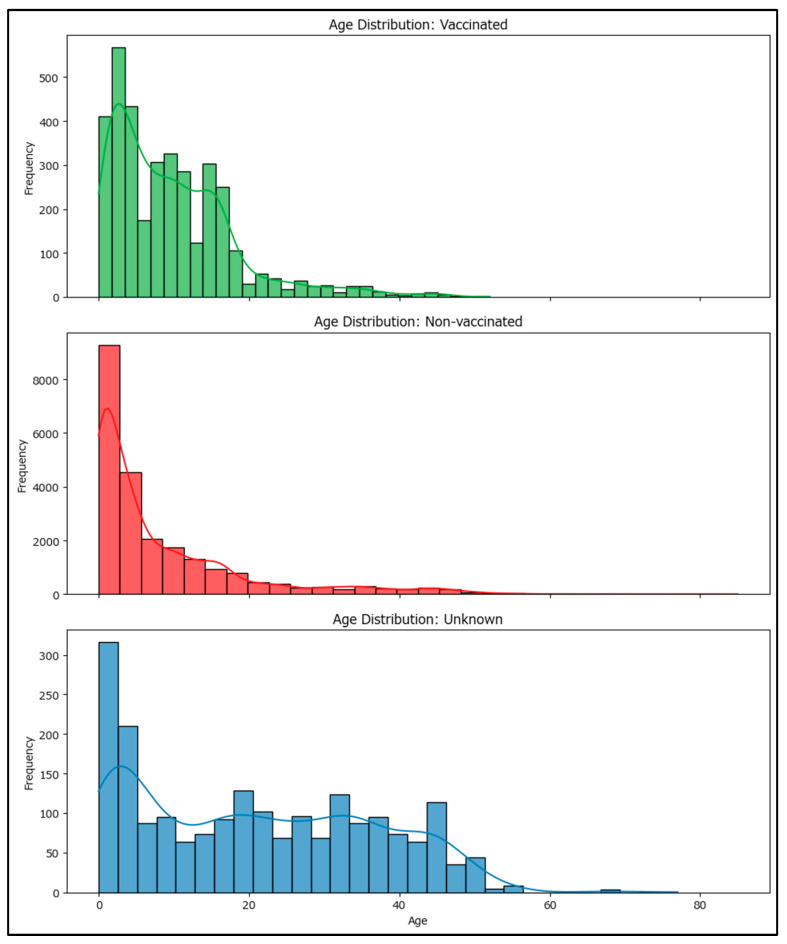
Age distribution by MMR vaccination status among the notified measles cases in Romania in 2020–2024 (vaccinated = vaccinated with at least one MMR dose).

The mean age for the vaccinated group is approximately 13.76 years, while the mean age for the non-vaccinated group is about 8.31 years. This indicates that, on average, individuals who are vaccinated and develop measles tend to be older than those who are not vaccinated and develop the disease. This provides insight into how recent vaccination campaigns may be less successful compared to the pre-COVID-19 period, with younger age groups becoming more vulnerable (Figure 2).

### 3.1. Results by Age Group and MMR Vaccination Status

Vaccination status segregated by relevant age groups is plotted in Figure 2. Most cases occurred in the 1–4 years age group. The non-vaccinated proportion is high across all age groups, with a notable proportion having only one MMR dose in the 1–4 years group, consistent with the need for a second dose for a better protection profile. The vaccination status is not consistent across the country, with counties exhibiting significant differences due to different demographics, social, and economic disparities, as well as unequal impact from measles outbreaks. Darker colors suggest that more vaccinated cases occurred in that specific age group and county. For example, counties with more cluster-related measles cases, such as Alba or Brașov, had more children under one year old who, although swiftly vaccinated in the context of a cluster setting, still developed the disease.

**Figure 3:**
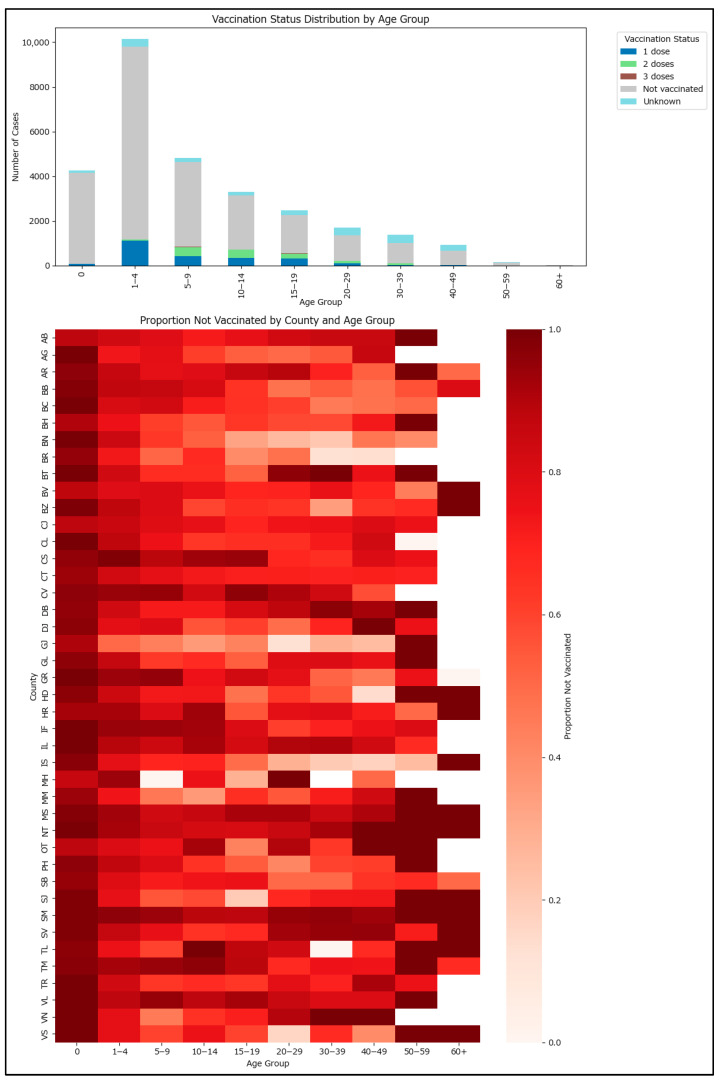
Age distribution by MMR vaccination status among the notified measles cases in Romania in 2020–2024 (countrywide—above, segregated by county—below).

The highest-risk age groups are those under one year and those older than 55–60, who may not have received the initial measles vaccine introduced in Romania in 1979 (Figure 3).

Statistical testing using the Kruskal–Wallis test revealed highly significant differences in hospitalization duration across age groups (<1, 1–4, 5–9, 10–19, 20–39, 40–59, >60 years) (statistic value 111.5157, *p*-value < 0.0001) and vaccination statuses (unknown, 0, 1, 2, or 3 doses) (statistic value 31.92425, *p*-value < 0.0001), which confirm that both age and vaccination status are associated with differences in the length of hospital stay for the cases in the dataset.

**Figure 4:**
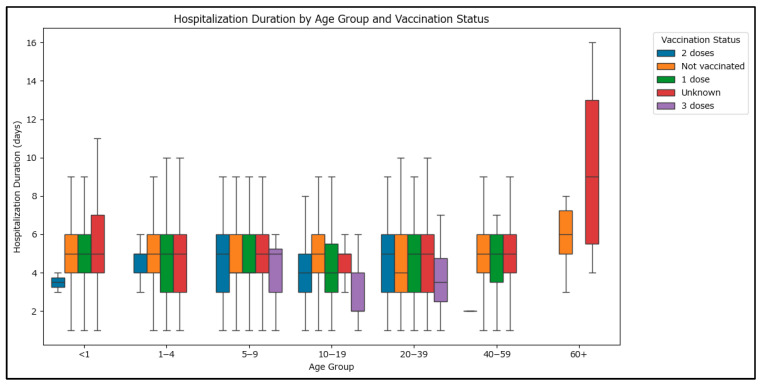
Hospitalization duration by age group and MMR vaccination status for the notified measles cases in Romania in 2020–2024.

As seen in Figure 4, higher hospitalization durations can be observed in the >60 age group. Although the difference in hospitalization duration between those vaccinated and non-vaccinated is statistically significant, the Mann–Whitney U test reveals that the effect size (r) is approximately −0.05, indicating a very small difference in hospitalization duration between the groups, in favor of the vaccinated group.

### 3.2. Results by Symptoms and Complications Prevalence

Vaccinated cases may exhibit different profiles for both symptoms and complications during disease development. To assess whether there is a difference, cases with at least one MMR dose were compared to those not vaccinated. Fever and rash are both mandated by the measles case definition, as mentioned in the measles surveillance methodology and; as such, only rash has been included as a witness test with a prevalence of 1 or very close to 1.

(a) Symptoms Prevalence

Measles can manifest through multiple clinical features such as fever, rash, coryza, cough, conjunctivitis, lymphadenopathy, arthritis, or arthralgia. Arthritis has a very low prevalence with only 12 total cases reported—10 non-vaccinated and 2 vaccinated with one MMR dose.

The frequency and proportion of each symptom were calculated for both vaccinated and non-vaccinated groups in Table 2. To quantify the magnitude of differences, the effect sizes such as odds ratios and Cohen’s h were computed. Most symptoms were common in both groups, but symptoms like cough and conjunctivitis were significantly less frequent among vaccinated cases. The effect sizes (Cohen’s h) for these symptoms indicate a moderate to strong difference, with negative values showing lower prevalence in vaccinated individuals. Odds ratios below 1 for these symptoms further confirm a protective effect of vaccination. Statistical tests (chi-square) also showed highly significant differences for several symptoms, especially cough and conjunctivitis. These findings suggest that vaccination is associated with a reduced risk of severe or additional symptoms, highlighting its public health benefit in mitigating disease severity.

**Figure 5:**
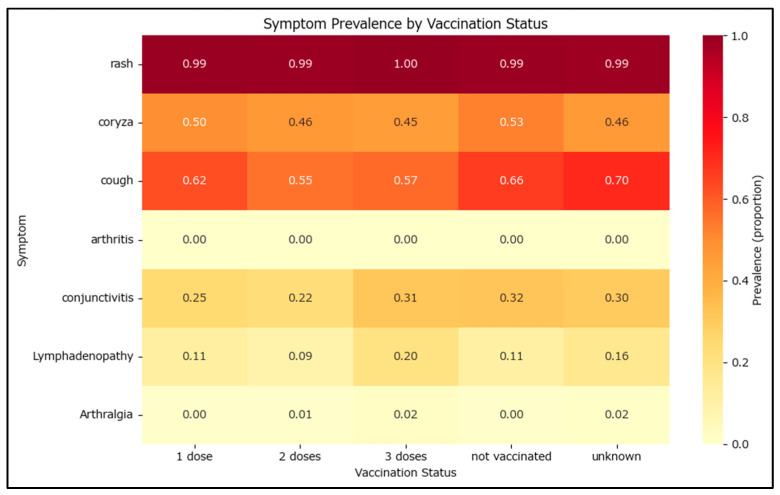
Symptom prevalence by MMR vaccination status.

The symptom prevalence generally decreases as the number of MMR vaccine doses increases. The most significant changes occur for coryza (5.6% decrease from non-vaccinated to one dose and then a further 8% decrease after the second MMR dose) and cough (6.4% decrease from non-vaccinated to one dose and then a further 11.3% decrease after the second MMR dose) (Figure 5).

(b) Complications Prevalence

**Figure 6:**
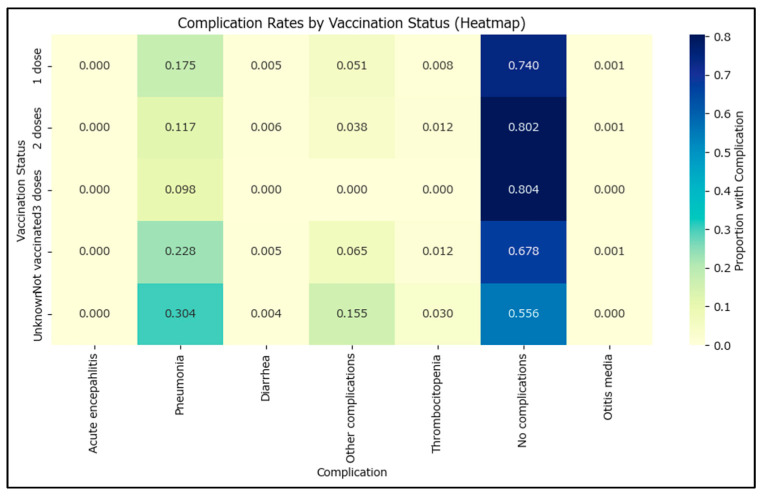
Complication rates by MMR vaccination status.

Complications often associated with measles are diarrhea, otitis, or pneumonia (Figure 6). The comparison of complications between vaccinated and non-vaccinated cases reveals several important findings. Pneumonia and other complications are significantly less common among vaccinated individuals, with odds ratios well below 1 and confidence intervals that do not include 1 for the ‘other complications’ (e.g., severe dehydration) category (Table 3). The effect size (Cohen’s h) for pneumonia is moderate, and the difference is highly statistically significant. The prevalence of no complications is higher in vaccinated cases, with an odds ratio above 1 and a confidence interval that does not include 1, indicating a strong protective effect of vaccination. For other complications such as acute encephalitis, diarrhea, thrombocytopenia, and otitis media, the odds ratios are close to 1 and the confidence intervals include 1, suggesting no significant difference between groups. These results emphasize that vaccination is associated with a reduced risk of certain complications, particularly pneumonia and other complications, and an increased likelihood of having no complications.

Going from non-vaccinated to one MMR dose shows a 9.1% higher prevalence of no complications, with two doses increasing the no complications prevalence up to 80% of the measles cases.

Subacute sclerosing panencephalitis, in the form of acute encephalitis, was recorded for seven cases (six non-vaccinated, one with an incomplete vaccination course—one MMR dose at the age of 11). This amounts to a subacute sclerosing panencephalitis rate of 3.19 cases/100.000 measles cases, which is consistent with findings from other countries such as Papua New Guinea (2.9/100,000) [17] or India [18].

A logistic regression analysis was conducted to explore the relationship between the time since the last vaccination and the likelihood of experiencing clinical complications. The model found that the coefficient for the interval (in days) since the last vaccine was very close to zero and not statistically significant (*p* = 0.52). This suggests that, within the analyzed dataset, the time elapsed since the last vaccination does not have a significant effect on the odds of developing clinical complications. The intercept was significant, but the interval itself was not a meaningful predictor in this context.

Further investigation was performed using time-to-event among vaccinated cases, using days as the measuring unit between the vaccination and measles onset. Only 3171 cases which had both dates available have been included in the analysis, as the rest of the 503 vaccinated cases had an unknown date of vaccination (most commonly, patients/their parents declared they had been vaccinated age-appropriately but are unable to provide proof, with the patient also missing from the National Electronic Vaccination Register).

**Figure 7:**
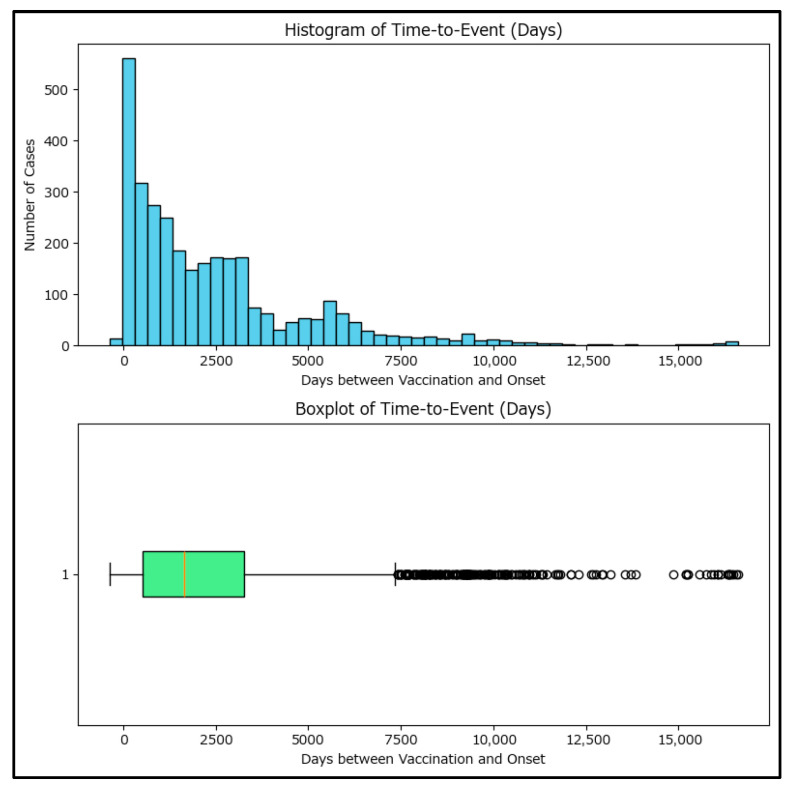
Histogram and boxplot of time-to-event in days between vaccination and measles onset for the 3171 eligible cases.

The plot (Figure 7) shows a wide range of time-to-event values, with some cases occurring shortly after vaccination and others many years later, with a median of 1651 days. The representations also reveal a right-skewed distribution with some negative values, indicating possible data entry errors or cases where onset preceded vaccination.

**Figure 8:**
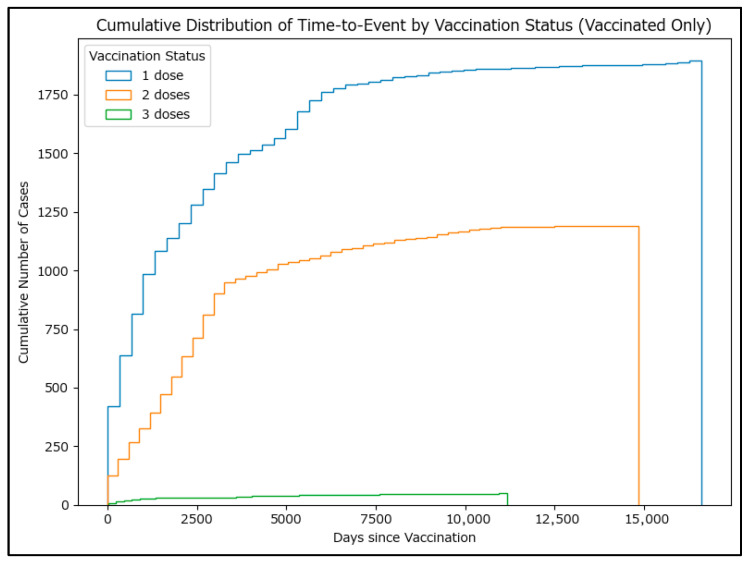
Cumulative histograms for time-to-event by MMR vaccination status (vaccinated only).

Cumulative histograms (as a proxy for survival curves, Figure 8) also show that most cases occurred several years after vaccination, but a small number occurred within the first year. These findings suggest that while most cases occur long after vaccination, there are exceptions that may warrant further investigation. Although genotyping is strongly recommended for cases where measles occurs shortly after MMR vaccination, in the few cases where genotyping has been performed (88 positive genotyping results during the period analyzed), vaccinal A-type-derived strains of measles have not been identified.

Negative time-to-event cases were extracted and each case was explored to understand the data incongruity. Thirty-one cases were identified with the date of onset preceding the date of MMR vaccination, 17 with one MMR dose and 14 with two MMR doses. The cases were evenly spread across counties and mostly affected young children (18 cases aged under 10 years), with the onset preceding the vaccination usually by two days, which supports their validity, as the cases were vaccinated while exhibiting coughing, coryza, or low-grade fever before developing the characteristic rash, which the practitioner did not consider contraindications to the MMR vaccine.

### 3.3. Results by Hospitalization

As Romania no longer has herd immunity for measles, and measles has been shown to exhibit significantly different measles-related hospitalization rates in such circumstances, this issue is treated separately. Most of the reported data originate from hospitals, indicating that both vaccinated and non-vaccinated patients were more likely to be treated in hospital settings than at home. Measles testing was predominantly performed for individuals presenting for hospital consultations. Hospitalization for measles as an isolation measure is no longer mandatory in Romania.

**Figure 9:**
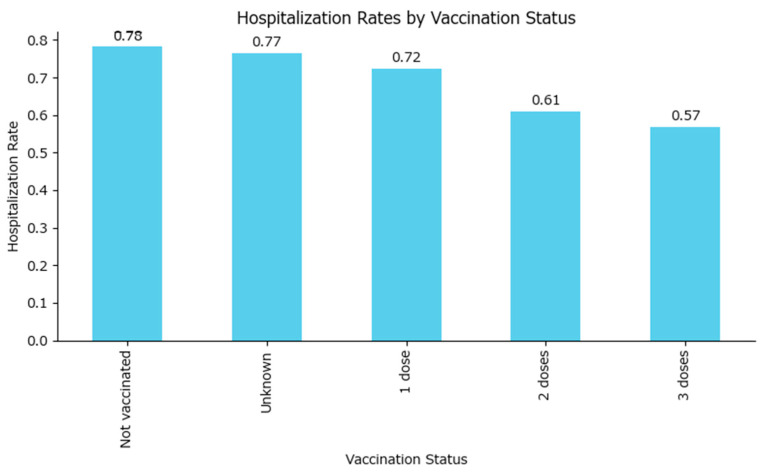
Measles hospitalization rates by MMR vaccination status.

The hospitalization rate was highest among the non-vaccinated group (approximately 78%), similar to those with an unknown vaccine status, while it was lower in all vaccinated groups in a dose-dependent manner: one MMR dose—72%, two MMR doses—61%, and three MMR doses—57% (Figure 9).

A chi-square test was performed to assess the significance of differences in hospitalization rates across vaccination status groups.

The contingency table (Table 4) shows the counts of hospitalized and non-hospitalized cases for each vaccination status. The chi-square statistic is 236.34 (4 degrees of freedom), and the *p*-value is extremely small (*p* < 0.0001), indicating a highly significant difference in hospitalization rates between vaccination status groups (unknown, 0, 1, 2, or 3 doses). This confirms that the vaccination status is strongly associated with the likelihood of hospitalization among measles cases, which will be explored using odds ratios.

Using the non-vaccinated group as the reference, the odds ratio for hospitalization comparing vaccinated to non-vaccinated individuals have been plotted in Table 5, with an overall value of 0.60. This means that vaccinated individuals, even if they were vaccinated with only one MMR dose, have about 40% lower odds of being hospitalized compared to those who are not vaccinated.

All odds ratios among vaccinated cases are less than 1 (including the confidence intervals), indicating that individuals with any number of vaccine doses have lower odds of hospitalization compared to those who are not vaccinated. The protective effect appears stronger with more doses, although the confidence interval also increases with the number of doses as the number of measles cases among those vaccinated decreases, as the vaccinated population is better safeguarded against the disease in the first place.

A multivariable logistic regression has been performed to model the probability of hospitalization as a function of vaccination status (non-vaccinated and fully vaccinated with 2–3 doses), age, and sex. The coefficients are shown in Table 6.

All predictors were statistically significant except for the unknown vaccination status, which was marginally non-significant. These findings suggest that both vaccination and younger age are protective against hospitalization, and there are notable differences by sex. The model fit and coefficients are robust, but possible residual confounding and the high proportion of missing data in some variables need cautious interpretation of the results.

Increasing age is associated with a lower likelihood of hospitalization (negative coefficient, highly significant). Male gender (relative to female) is associated with a lower likelihood of hospitalization (negative coefficient, significant). Having two or three vaccine doses is associated with a lower probability of hospitalization compared to the reference group (statistically significant negative coefficients for these categories). Unknown vaccination status and non-vaccinated show positive coefficients, indicating a higher risk compared to the reference group.

**Figure 10:**
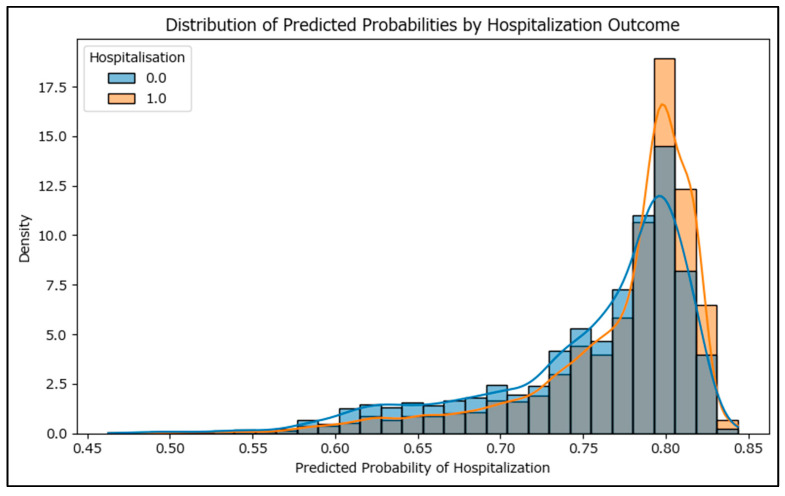
Distribution of predicted probability by hospitalization outcome.

To assess the performance of the logistic regression model, the receiver operating characteristic curve was used, and with an area under curve of 0.595 (Figure S1), the model has, at most, only a fair effectiveness in predicting hospitalization based on vaccination status, age, and sex. The distribution of predicted probabilities from the logistic regression model is visualized as a histogram (Figure 10), separated by actual hospitalization outcome. Ideally, the predicted probabilities for hospitalized cases should cluster toward higher values, while those for non-hospitalized cases should cluster toward lower values. The low degree of separation between these distributions reflects the model’s less-than-ideal discriminative power.

#### Best-Case/Worst-Case Scenario Hospitalization Analysis for Unknown MMR Vaccine Status

As the dataset contains a significant number of cases with unknown vaccination status, mostly due to the inability to retrieve data predating the electronic vaccination register (2006 cases—6.88% of the total) and the risk of affecting the results, multiple bias analysis scenarios were conducted.

The hospitalization rate for vaccinated cases is 68.3%, for non-vaccinated cases 78.2%, while for cases with unknown vaccination status, the hospitalization rate is 76.6%.

In the best-case scenario (all unknowns treated as vaccinated), the combined hospitalization rate for the vaccinated group rises to 71.2%. In the worst-case scenario (all unknowns treated as non-vaccinated), the combined rate for the non-vaccinated group becomes 78.1%.

This analysis demonstrates that the potential bias from unknown vaccination status could shift the estimated hospitalization rate for the vaccinated group upward, but the difference between best-case and worst-case scenarios is modest. The relationship between vaccination status and hospitalization remains robust, with vaccinated cases consistently showing a lower hospitalization rate than non-vaccinated cases, even when accounting for uncertainty in the unknown group.

### 3.4. Limitations

Since our data is limited to notified cases of measles, the findings and associations presented should not be construed as direct causal effects or as representative measures of vaccine effectiveness. Such limitations prevent inference regarding overall measles incidence or the general population’s vaccine effectiveness. Instead, the study aims to assess the relative burden among reported cases and emphasize the beneficial effect of the MMR vaccination even when the vaccinated person develops the disease.

Data accuracy remains a significant concern, as the potential for misreporting cannot be disregarded. This may result from inaccuracies in data entry—for example, patients might report receiving vaccinations that did not occur—or from errors in data management due to the large-scale database overseen by an extensive team. Even with continuous corrections, inaccuracies may persist, particularly during outbreaks when workload increases substantially.

Underreporting or case invalidation resulting from improper collection or handling of biological specimens presents a significant challenge, as the numerous procedural steps required to accurately include a measles-compatible case in the dataset introduce multiple potential limitations. For example, minorities are less likely to report to a doctor unless the symptoms become severe or even life-threatening, and even then, there may be communication barriers or lower-than-ideal acceptability for treatment or specimen collection. Physicians may refrain from reporting suspected measles cases even though it is a mandatory requirement, as the process involves bureaucratic steps that demand additional time and effort. This can be particularly challenging during working hours when practitioners have limited resources to allocate to administrative obligations.

## 4. Discussion

Measles remains a critical public health issue in Romania, as the ever-dropping MMR vaccine coverage rates have led to a loss of herd immunity, which, as previously shown, has a negative effect beyond amplifying the disease’s spread patterns, which is increased disease burden through increased hospitalization rates. The measles-related hospitalization rate is very high in Romania (over 70%) compared to other countries such as Israel (6.6% hospitalization rate among the pediatric population) [19] or the US, with a hospitalization rate of 13.3% (171 of 1356 cases) in 2025 [20]. This does not suggest a more severe measles profile in Romania but rather another concern spread worldwide, severe measles underreporting. The lower-than-expected notification rate has been documented as it had already raised concerns even before COVID-19 [21]. As measles cases continue to be on the rise in the US throughout 2025, the CDC also expressed its concerns regarding a large amount of cases which may go unreported [22]. Although widespread, such an issue is very difficult to quantify, and instead we are given glimpses through indirect data. A thought exercise based on the data analyzed in this article, a dataset with 20,856 hospitalized cases, under a 20% optimistic hospitalization rate scenario would lead to more than 100,000 measles cases over the 5-year period (2020–2024). In this scenario, two out of three cases may have gone unreported, which is not outside the realm of possibility, as public health difficulties go well beyond vaccination programs, confounded by the already low trust in healthcare specialists [23], that was hurt even more by the COVID-19 pandemic [24], thereby artificially lowering healthcare accessibility through psycho-emotional barriers. Vulnerable populations, such as Roma communities [25], which are prevalent in Romania, become even more vulnerable by avoiding visiting the doctor unless the measles symptoms become severe, thereby lowering the chances of a favorable outcome.

Vaccines from the national vaccination schedule in Romania face several limiting factors in reaching the target population. Roma communities, compounded by lower education levels, are more difficult to reach out to in the absence of a competent mediator and, due to cultural norms, also tend to aggregate in more aggressive and harder-to-control clusters. This issue is made even worse by the chronic underfunding of the healthcare system, with general practitioners being less compelled to address smaller settlements. A considerable number of rural areas lack any kind of doctor to whom the population can address. Political turmoil has also played a significant role in reducing vaccine coverage, especially for MMR and HPV, with at least one major political party submitting impeachment motions and promoting on social media claims that MMR and HPV vaccination campaigns were ‘too aggressive’ or that MMR vaccines have ‘hidden risks’ in the context of an overly aggressive measles epidemic. The Romanian National Order of Physicians publicly expressed its disdain against such non-scientifically based ideas in the public space and once again raised concerns about the very low level of health education in Romania, which has also faced challenges in being implemented in schools [26].

With the ongoing war in Ukraine and seaside tourism in Bulgaria, measles importation from neighboring countries has also been a concern. In our dataset, only 138 cases (0.0047%) were imported from other countries; these cases do not exhibit clustering along the counties near the border instead they were evenly distributed throughout the country, with most common probable countries of infection being the United Kingdom of Great Britan (15 cases), Italy (11 cases), or Germany (10 cases).

Previous approaches involving mandatory hospitalization for specific infectious diseases with a high-risk epidemiological profile, such as scarlet fever or measles, provided better control and surveillance data but at a very high economic and social cost. However, the problem of unnecessary hospitalizations in Romania’s public hospitals remains controversial [27].

As COVID-19 negatively modeled the population’s view on immunizations due to its inability to provide herd protection as, in its case, the classical herd immunity did not readily apply, the effects bled into other immunization schedules which do provide classical herd immunity, such as those for measles or polio [28,29]. In Romania, although the notified measles cases remained high throughout 2023 and 2024, the MMR vaccine distrust impediment continued to worsen. The ECDC noted that Romania had the sharpest decline in coverage for both MMR vaccines in 2020–2023 (−9% for MMR1 dose and −13% for MMR2 dose) [30]. In such circumstances, increasing data transparency and disseminating medical research results in accessible formats [22] remain the only tangible options to increase MMR vaccine coverage for the time being, as enforcing a mandatory vaccination schedule would most likely lead to social turmoil. Increasing health literacy is desirable before making the vaccine compulsory, as previously taught by Germany’s situation, where the mandatory vaccine approach led to a worsening of the vaccine coverage compared to the liberal approach [31].

Although measles occurs infrequently among older adults, they tend to get hospitalized more often and have a significant longer hospital stay, which warrants consideration of including the MMR vaccine in the modern vision of lifelong or adult immunization [32]. Romania is already offering the MMR vaccine for adults with bone marrow transplants [10], but revising the risk categories to which it is offered and how it is promoted may yield better results. The MMR vaccine in adults has already been proven to be safe [33] and may also be safe for adults undergoing immunosuppressive therapy [34].

The findings of this study should be integrated in the action plan for the elimination of measles adopted in Romania in 2022, which has a long-term vision up to 2030, as they provide additional perspectives on how measles evolved in the first years since its implementation and how it can be improved upon.

## 5. Conclusions

As the measles threat continues throughout the European region, exacerbated by the continuously lowering vaccination rates, especially in Romania, where herd immunity is no longer an obtainable goal in the short term, our efforts should go toward emphasizing the individual benefits of immunization. To support this, the analysis on the Romania’s 2020–2024 measles cases shows that MMR vaccines provide protection beyond primary disease prevention, as they reduce disease burden by reducing the hospitalization rate among those affected by measles by more than 50% for complete, two-dose MMR immunization, as well as improving clinical outcomes.

## Figures and Tables

**Table 1:** Distribution of living environment by MMR vaccination status among notified measles cases in Romania in 2020–2024.

Living Environment	Rural	Urban
Vaccination Status		
1 MMR dose	57.8574%	42.1426%
2 MMR doses	60.4441%	39.5559%
3 MMR doses	50.9804%	49.0196%
Non-vaccinated	54.2675%	45.7325%
Unknown	45.1323%	54.8677%

**Table 2:** Statistical test and effect size for each symptom comparing between the vaccinated cases with one, two, or three MMR doses against those non-vaccinated.

Symptom	Vaccinated Proportion	Non-Vaccinated Proportion	Odds Ratio	CI Lower	CI Upper	Cohen’s h	Chi2	*p*-Value
rash	0.9931	0.990498	1.380691	0.911837	2.090623	0.028941	2.064438	0.15077
coryza	0.486613	0.52578	0.8549	0.797099	0.916894	−0.07836	19.13383	1.22 × 10^−5^
cough	0.598951	0.66205	0.762351	0.709579	0.819047	−0.13084	54.91486	1.26 × 10^−13^
arthritis	0.001104	0.000469	2.356954	0.750093	7.406063	0.023164	1.285128	0.256948
conjunctivitis	0.23958	0.317496	0.677275	0.624522	0.734483	−0.17419	89.17847	3.61 × 10^−21^
lymphadenopathy	0.101573	0.10542	0.959384	0.85468	1.076915	−0.01263	0.454494	0.500209
arthralgia	0.005796	0.004346	1.335549	0.833899	2.138977	0.020465	1.156722	0.282146

**Table 3:** Complication effect sizes and statistical tests summaries comparing between vaccinated and non-vaccinated groups.

	Complication	Vaccinated Proportion	Non-Vaccinated Proportion	Odds Ratio	CI Lower	CI Upper	Cohen h	Chi2	*p*-Value
0	Acute encephalitis	0.000276	0.000341	0.809636	0.10123	6.475445	−0.0037	0	1
1	Pneumonia	0.155672	0.228098	0.623937	0.567475	0.686016	−0.18467	96.01479	1.14 × 10^−22^
2	Diarrhea	0.005244	0.005156	1.017219	0.626335	1.652046	0.001228	0	1
3	Other complications	0.046646	0.064982	0.704029	0.598194	0.828588	−0.08014	17.68836	2.6 × 10^−5^
4	Thrombocytopenia	0.009384	0.011803	0.793131	0.554512	1.134435	−0.02366	1.41197	0.23473
5	No complications	0.761248	0.678285	1.512295	1.39443	1.640124	0.18518	100.49	1.19 × 10^−23^
6	Otitis media	0.000828	0.000937	0.883199	0.264211	2.952346	−0.00369	0	1

**Table 4:** Contingency table: Hospitalization (yes/no) by MMR vaccination status.

Hospitalization	No	Yes
Vaccination Status		
1 dose	667	1737
2 doses	476	743
3 doses	22	29
Non-vaccinated	5121	18,347
Unknown	470	1536

**Table 5:** Hospitalization rates and odds ratios by MMR vaccination status groups (one, two, or three doses, or unknown status), using non-vaccinated group as the reference.

Vaccination Status	Hospitalization Rate	Odds Ratio for Hospitalization	CI Lower	CI Upper	Log-Odds	Std Error
1 dose	72.25%	0.726882	0.661335	0.798924	−0.31899	0.048216
2 doses	60.95%	0.435684	0.386739	0.490823	−0.83084	0.060799
3 doses *	56.86%	0.367930	0.211214	0.640925	−0.99986	0.283173
Unknown	76.57%	0.912185	0.818916	1.016078	−0.09191	0.055032

* The results for the three MMR doses group are exploratory only as the group is very tiny compared to the dataset.

**Table 6:** Statistical results of the multivariable logistic regression modeling the probability of hospitalization.

	Coef.	Std. Err.	z	*p* > |z|	Confidence Intervals [0.025, 0.975]
const	1.174542	0.049335	23.8077	2.8 × 10^−125^	[1.0778, 1.2712]
Age	−0.01952	0.001195	−16.3304	6 × 10^−60^	[−0.021, −0.017]
Gender_bin	−0.11957	0.028241	−4.23375	2.3 × 10^−5^	[−0.174, −0.064]
Vax_2 doses	−0.41964	0.07488	−5.60417	2.09 × 10^−8^	[−0.566, −0.272]
Vax_3 doses	−0.55741	0.291168	−1.91438	0.055571	[−1.128, 0.0132]
Vax_Non-vaccinated	0.334916	0.048436	6.914603	4.69 × 10^−12^	[0.2399, 0.4298]
Vax_Unknown	0.510516	0.0726	7.031926	2.04 × 10^−12^	[0.3682, 0.6528]

## Data Availability

Data are available upon request through the corresponding authors.

## Supplementary Materials

The following supporting information can be downloaded at: https://www.mdpi.com/article/10.3390/epidemiologia6040063/s1, Figure S1: ROC curve for the performance of the multivariable logistic regression model for the probability of hospitalization.

## References

[1] ECDC Immunisation and Vaccines. https://www.ecdc.europa.eu/en/immunisation-and-vaccines.

[2] (2025). European Centre for Disease Prevention and Control Vaccine Effectiveness. https://www.ecdc.europa.eu/en/infectious-disease-topics/related-public-health-topics/immunisation-and-vaccines/vaccine.

[3] Rademacher J., Therre M., Hinze C.A., Buder F., Bohm M., Welte T. (2024). Association of respiratory infections and the impact of vaccinations on cardiovascular diseases. Eur. J. Prev. Cardiol..

[4] Wilder-Smith A.B., Qureshi K. (2020). Resurgence of Measles in Europe: A Systematic Review on Parental Attitudes and Beliefs of Measles Vaccine. J. Epidemiol. Glob. Health.

[5] Neely S.R., Hao F. (2023). Breakthrough COVID-19 infections and perceived vaccine effectiveness. Vaccine.

[6] Novilla M.L.B., Goates M.C., Redelfs A.H., Quenzer M., Novilla L.K.B., Leffler T., Holt C.A., Doria R.B., Dang M.T., Hewitt M. (2023). Why Parents Say No to Having Their Children Vaccinated against Measles: A Systematic Review of the Social Determinants of Parental Perceptions on MMR Vaccine Hesitancy. Vaccines.

[7] Ministry of Health, Romania (2022). Order no 3975/2022 Technical Norms for Implementing the National Public Health Programs (The National Immunization Schedule). The Official Monitor. https://lege5.ro/Gratuit/geztcmzygu2di/calendarul-national-de-vaccinare-ordin-3975-2022?dp=guytcobsgiztqmq.

[8] Ministry of Health, Romania (2023). Order no 4128/2023 Declaring a Measles Epidemic on the Territory of Romania and Approving Measures to Limit the Spread of the Epidemic. The Official Monitor. https://legislatie.just.ro/public/DetaliiDocument/276996.

[9] (2024). National Institute for Public Health Romania, Infectious Disease Under Surveillance Analysis—Report for 2023. https://insp.gov.ro/download/analiza-bolilor-transmisibile-aflate-in-supraveghere-raport-pentru-anul-2023/.

[10] Stanescu A., Ruta S.M., Cernescu C., Pistol A. (2024). Suboptimal MMR Vaccination Coverages-A Constant Challenge for Measles Elimination in Romania. Vaccines.

[11] Altindag O., Greve J., Tekin E. (2025). Impact of Measles, Mumps, and Rubella Vaccination on Hospitalizations and Human Capital: Evidence from Copenhagen School Health Records. Vaccines.

[12] (2024). National Institute for Public Health Romania, Measles and Rubella Surveillance Methodology. https://insp.gov.ro/centrul-national-de-supraveghere-si-control-al-bolilor-transmisibile-cnscbt/metodologii/.

[13] COMMISSION IMPLEMENTING DECISION (EU) 2018/945 of 22 June 2018 on the Communicable Diseases and Related Special Health Issues to Be Covered by Epidemiological Surveillance as Well as Relevant Case Definitions. https://eur-lex.europa.eu/eli/dec_impl/2018/945/oj/eng.

[14] Ekmekci B., McAnany C.E., Mura C. (2016). An Introduction to Programming for Bioscientists: A Python-Based Primer. PLoS Comput. Biol..

[15] Greenberg-Worisek A., Ferede L., Balls-Berry J., Marigi I., Valentin Mendez E., Bajwa N., Ouk M., Orellana M., Enders F. (2020). Differences in Electronic Personal Health Information Tool Use Between Rural and Urban Cancer Patients in the United States: Secondary Data Analysis. JMIR Cancer.

[16] National Institute for Public Health (2024). VACCINAREA Analiză de Situație. https://insp.gov.ro/wp-content/uploads/2024/04/Analiza-de-situatie-VACCINARE-2024.pdf.

[17] Manning L., Laman M., Edoni H., Mueller I., Karunajeewa H.A., Smith D., Hwaiwhanje I., Siba P.M., Davis T.M. (2011). Subacute sclerosing panencephalitis in papua new guinean children: The cost of continuing inadequate measles vaccine coverage. PLoS Negl. Trop. Dis..

[18] Mishra B., Kakkar N., Ratho R.K., Singhi P., Prabhakar S. (2005). Changing trend of SSPE over a period of ten years. Indian J. Public Health.

[19] Stein-Zamir C., Shoob H., Abramson D. (2023). Measles clinical presentation, hospitalization and vaccination status among children in a community-wide outbreak. Vaccine.

[20] CDC Measles Cases in 2025 CDC. https://www.cdc.gov/measles/data-research/index.html.

[21] Mette A., Reuss A.M., Feig M., Kappelmayer L., Siedler A., Eckmanns T., Poggensee G. (2011). Under-reporting of measles: An evaluation based on data from north rhine-westphalia. Dtsch. Arztebl. Int..

[22] Bendix A. (2025). CDC says measles cases are most likely underreported as outbreak swells in Texas. NBC NEWS: Online.

[23] Toth C. (2020). Repertoires of Vaccine Refusal in Romania. Vaccines.

[24] Perlis R.H., Ognyanova K., Uslu A., Lunz Trujillo K., Santillana M., Druckman J.N., Baum M.A., Lazer D. (2024). Trust in Physicians and Hospitals During the COVID-19 Pandemic in a 50-State Survey of US Adults. JAMA Netw. Open.

[25] Foldes M.E., Covaci A. (2012). Research on Roma health and access to healthcare: State of the art and future challenges. Int. J. Public Health.

[26] Arambescu D. (2025). Colegiul Medicilor, reacție acidă după moțiunea AUR antivaccin HPV: “Medicina se bazează pe dovezi, nu pe presupuneri”. Libertatea.

[27] Jullien S., Mateescu I., Brinzac M.G., Dobocan C., Pop I., Weber M.W., Butu C., Carai S. (2023). Unnecessary hospitalisations and polypharmacy practices in Romania: A health system evaluation for strengthening primary health care. J. Glob. Health.

[28] Morens D.M., Folkers G.K., Fauci A.S. (2022). The Concept of Classical Herd Immunity May Not Apply to COVID-19. J. Infect. Dis..

[29] Larson H.J., Gakidou E., Murray C.J.L. (2022). The Vaccine-Hesitant Moment. N. Engl. J. Med..

[30] European Centre for Disease Prevention and Control Measles—Annual Epidemiological Report for 2024. https://www.ecdc.europa.eu/sites/default/files/documents/MEAS-AER-2024-Report.pdf.

[31] Hollan M. (2012). Compulsory vaccination, the constitution, and the hepatitis B mandate for infants and young children. Yale J. Health Policy Law Ethics.

[32] Naranjo L., Dominguez E., Rodriguez C., Chandler R., Belen Arauz A., Barahona de Mosca I., Hernandez T., Coto F., Ramirez Chavez J., Sandoval N. (2022). Adult immunization practices, challenges and opportunities in Central America and the Caribbean: Advisory board proceedings. Hum. Vaccin. Immunother..

[33] Ami N., Eyal N., Asaf B., Chen A., Adi B., Drorit A., Neta P., Hajar D., Stav R., Eli S. (2021). Safety of measles, rubella and mumps vaccines in adults: A prospective cohort study. J. Travel. Med..

[34] Worth A., Waldman R.A., Dieckhaus K., Rothe M.J. (2020). Art of prevention: Our approach to the measles-mumps-rubella vaccine in adult patients vaccinated against measles before 1968 on biologic therapy for the treatment of psoriasis. Int. J. Womens Dermatol..

