# Supplementary material for: Positive Impact of Vaccinal Status Among Notified Measles Cases in Romania in 2020–2024
Source: Epidemiologia (Basel). 2025 Oct 11;6(4):63. doi: 10.3390/epidemiologia6040063 (PMC12550983; doi:10.3390/epidemiologia6040063)
Supplement: Supplementary file 1 [file epidemiologia-06-00063-s001.zip › epidemiologia-3840223-supplementary.pdf]

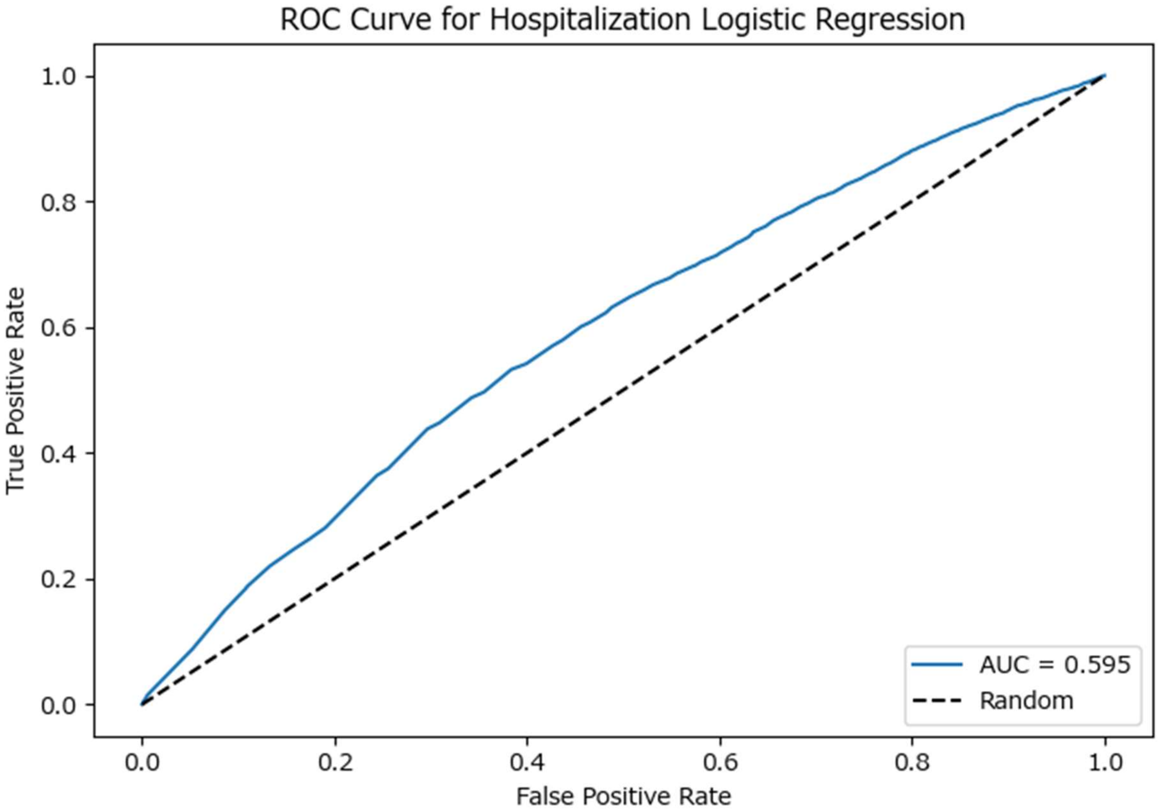

Figure S1. ROC curve for the performance of the multivariable logistic regression model for the probability of hospitalization.
